# Editorial: The Emerging Role for PP2A in Hematologic Malignancies

**DOI:** 10.3389/fonc.2016.00195

**Published:** 2016-08-31

**Authors:** Peter P. Ruvolo

**Affiliations:** ^1^Department of Leukemia, University of Texas MD Anderson Cancer Center, Houston, TX, USA

**Keywords:** PP2A, leukemia, signal transduction, chemotherapy, microRNA, cellular inhibitors

**The Editorial on the Research Topic**

**The Emerging Role for PP2A in Hematologic Malignancies**

While we strive to understand the role of kinases in signal transduction pathways that mediate leukemogenesis, drug resistance, and homeostasis of leukemia cells, the role of the protein phosphatases that are also critical in these cascades is under studied. The PP2A serine/threonine family of protein phosphatases contains many tumor suppressors, and these enzymes regulate a large number of survival molecules that are essential to leukemia cell survival. In this Research Topic, The Emerging Role for PP2A in Hematologic Malignancies, the authors review the current state of knowledge on PP2A role in leukemia and discuss the emergence of new therapeutic strategies to target PP2A for leukemia therapy.

A background in PP2A structure and function is provided by Haesen et al. in “The basic biology of PP2A in hematologic cells and malignancies.” This review introduces us to the subunits that comprise the PP2A heterotrimer – the catalytic C subunit and scaffold A subunit that make up the catalytic core and the regulatory B subunits that drive isoform specificity and function. The authors provide detailed information on the expression of these subunits in hematologic tissues as well as the various cellular proteins that regulate PP2A function including the various inhibitors such as SET that have been implicated in suppressing PP2A tumor suppressor function in many cancers. The review provides an excellent description of the post-translational modifications that control how the PP2A heterotrimer assembles.

In “Therapeutic Reactivation of Protein Phosphatase 2A in Acute Myeloid Leukemia,” Ramaswamy et al. cover strategies to re-activate PP2A for leukemia therapy. The authors offer a mechanistic insight into how the protein phosphatase overcomes suppression in the leukemia cells. The authors also discuss how expression of the various subunits that comprise the PP2A heterotrimer vary in AML patients compared to normal individuals, suggesting that there may be distinct sets of PP2A isoforms in the leukemia patients.

Oaks and Ogretmen discuss the role sphingolipids such as ceramide play in controlling PP2A activity in “Regulation of PP2A by Sphingolipid Metabolism and Signaling.” Ceramides are sphingolipids that are key modulators of leukemia cell survival, and the review discusses how ceramides regulate PP2A function directly and indirectly *via* the SET inhibitor. The review also discusses the biology of FTY-720, a sphingosine analog that has been proposed for therapy of leukemia.

In “PP2A: The Achilles Heal in Myelodysplastic Syndrome (MDS) with 5q Deletion,” Sallman et al. discuss mechanistic aspects of PP2A catalytic C alpha subunit deletion in MDS. The PP2A subunit C alpha (PPP2CA) gene is located on human chromosome 5q, and this chromosomal region is deleted in 15% of MDS patients. The review discusses lenalidomide therapy for MDS and importance of PP2A as a lenalidomide target and the ability of PP2A catalytic C alpha subunit to act as a resistance factor to the drug.

Ciccone et al. discuss PP2A as a therapeutic target in leukemia in “From the Biology of PP2A to the PP2A-Activating Drugs (PADs) for Therapy of Hematologic Malignancies.” This review discusses the development and current use of PADS that include FTY-720, OP449, and AALS (a FTY-720 analog). The review includes background on cellular inhibitors such as SET and the mechanistic studies that linked SET to BCR ABL in chronic myeloid leukemia and Ph+ acute lymphoblastic leukemia. As discussed in the review, many of the PADs being developed target the SET inhibitor.

Arriazu et al. continue discussions of PP2A as a therapeutic target with emphasis on AML in “Protein Phosphatase 2A as a Therapeutic Target in Acute Myeloid Leukemia.” The review provides a comprehensive look at the state of current knowledge of expression of the various PP2A subunits in AML. The authors also provide detailed information on mechanistic aspects of the various PP2A isoforms and cellular inhibitors that impact leukemia cell survival. The review also provides information on Cancerous Inhibitor of Protein Phosphatase 2A (CIP2A), a cellular inhibitor of PP2A that is not as well studied as SET.

Finally, Ruvolo discusses how microRNAs (miRs) control various PP2A subunits and PP2A regulators and the emerging role of PP2A as a regulator of miR expression in “The Interplay between PP2A and miRs in Leukemia.” Competition between PP2A subunits is an important component in regulating multiple PP2A subunits and miRs may play a role in this process. The review also discusses the role of the PP2A B55 alpha regulatory subunit in controlling MYC and also miRs that are not dependent on MYC regulation.

It is our hope that this Research Topic encourages greater interest in PP2A role as a potential tumor suppressor in leukemia. A better understanding of PP2A contribution in MDS, leukemia, and other hematologic malignancies will hopefully encourage development of PADs for clinical use for therapy and also inspire the creation of newer PADs that will hopefully activate the PP2A isoforms that prove to be true tumor suppressors.

## Author Contributions

The author confirms being the sole contributor of this work and approved it for publication.

## Conflict of Interest Statement

The author declares that the research was conducted in the absence of any commercial or financial relationships that could be construed as a potential conflict of interest.

